# Assessment of the Neuroprotective Effects of *Lavandula angustifolia* Extract on the Contusive Model of Spinal Cord Injury in Wistar Rats

**DOI:** 10.3389/fnins.2016.00025

**Published:** 2016-02-08

**Authors:** Gholamreza Kaka, Kayvan Yaghoobi, Shaghayegh Davoodi, Seyed R. Hosseini, Seyed H. Sadraie, Korosh Mansouri

**Affiliations:** ^1^Neuroscience Research Center, Baqiyatallah University of Medical SciencesTehran, Iran; ^2^Department of Anatomy, School of Medicine, Baqiyatallah University of Medical SciencesTehran, Iran; ^3^Department of Physical Medicine and Rehabilitation, Iran University of Medical SciencesTehran, Iran

**Keywords:** spinal cord injury (SCI), *Lavandula angustifolia*, neuroprotection, Basso Beattie and Bresnahan (BBB), glial fibrillary acidic protein (GFAP), somatosensory evoked potential (SEP)

## Abstract

**Introduction:** Spinal cord injury (SCI) involves a primary trauma and secondary cellular processes that can lead to severe damage to the nervous system, resulting in long-term spinal deficits. At the cellular level, SCI causes astrogliosis, of which glial fibrillary acidic protein (GFAP) is a major index.

**Objective:** The aim of this study was to investigate the neuroprotective effects of *Lavandula angustifolia* (Lav) on the repair of spinal cord injuries in Wistar rats.

**Materials and Methods:** Forty-five female rats were randomly divided into six groups of seven rats each: the intact, sham, control (SCI), Lav 100, Lav 200, and Lav 400 groups. Every week after SCI onset, all animals were evaluated for behavior outcomes by the Basso, Beattie, and Bresnahan (BBB) score. H&E staining was performed to examine the lesions post-injury. GFAP expression was assessed for astrogliosis. Somatosensory evoked potential (SEP) testing was performed to detect the recovery of neural conduction.

**Results:** BBB scores were significantly increased and delayed responses on sensory tests were significantly decreased in the Lav 200 and Lav 400 groups compared to the control group. The greatest decrease of GFAP was evident in the Lav 200 and Lav 400 groups. EMG results showed significant improvement in the hindlimbs in the Lav 200 and Lav 400 groups compared to the control group. Cavity areas significantly decreased and the number of ventral motor neurons significantly increased in the Lav 200 and Lav 400 groups.

**Conclusion:** Lav at doses of 200 and 400 mg/kg can promote structural and functional recovery after SCI. The neuroprotective effects of *L. angustifolia* can lead to improvement in the contusive model of SCI in Wistar rats.

## Introduction

There are ~200,000 spinal cord injuries (SCI) in the United States annually, and the vast majority are caused by car accidents (Sekhon and Fehlings, 2001). SCI can involve severe damage to the motor, sensory, and autonomic nervous systems and their function, which may lead to paraplegia, tetraplegia, or other severe disabilities (Margaret, 2008). The pathogenesis of SCI after the primary trauma plays an important role in initial tissue disruption, and the subsequent initiation of a series of secondary cellular processes beyond the injury site can lead to long-term spinal deficits and disabilities (Beattie et al., 2002; Dumont et al., 2002). Increased oxidative stress (Azbill et al., 1997), activation of redox transcription factors, and elevated expression of inflammatory mediators may be some of the most important factors (Popovich and Jones, 2003) in the promotion of secondary injury after SCI.

Astrogliosis is a cellular response that creates a barrier to axonal regeneration, and the major index of astrogliosis is marked upregulation of glial fibrillary acidic protein (GFAP; Bharne et al., 2013). Therapeutic strategies attempt to attenuate astrogliosis during the initial phase after SCI (Labombarda et al., 2011). SCI results in increased tissue oxidative stress and production of reactive oxygen species (ROS; Azbill et al., 1997), and antioxidant protection after SCI has improved outcomes in experimental animals (Kamencic et al., 2001).

There has been interest in finding natural agents that may help to prevent the inflammation and degeneration of neural cells in SCI. A well-known herbal drug that has demonstrated antioxidant effects is lavender, or *Lavandula angustifolia* Mill. (Lamiaceae). Commonly known in Iran as “Ostokhoddous,” *L. angustifolia* is a widely distributed aromatic herb (Omidbeigi, 2006). It has been used widely for nervous system problems in Iranian traditional medicine (Avicenna, 1991), and it has recently been demonstrated that it has important effects on the central and peripheral nervous systems, including anti-inflammatory, anti-apoptosis, antioxidant, antimutant, and neuroprotective effects (Kayvan Yaghoobi et al., 2015). Gas chromatography-mass spectrometry analysis extraction of *L. officinalis* L. from Urmia, Iran, showed totals of 60 and 100 compounds, respectively, in 96 and 70% ethanol solvent extractions (Saadatian et al., 2013). The most abundant constituents observed in ethanol 96% extraction included ethane 29.80%,methanecarboxylic acid (9.01%), p-Vinylguaiacol (4.45%), pentadecanoic acid (3.67%), and dimethylamine, N,N-Dimethyl methanesulfonamide (2.06%; Saadatian et al., 2013). Yuanyuan et al. identified 17 compounds in lavender from Xinjiang, China, with linalool (44.54%), geraniol (11.02%), lavandulyl acetate (10.78%), 3,7-dimethyl-2, 6-octadien-1-ol (10.35%), and isoterpineol (6.75%) as the main components (Yuanyuan et al., 2008). It is known that linalool is responsible for important therapeutic effects (Peana et al., 2003, 2004). Each of these constituents can vary significantly in oils derived from different cultivars, and these variations can affect the medical properties; therefore, this study aimed to assess the effect of *L. angustifolia* extract from Iran on SCI. We hypothesized that *L. angustifolia* may play a role in preventing the harmful effects and neural damage triggered by SCI, and may promote axonal regeneration.

## Materials and methods

### Subjects

Adult female Wistar rats (Pastor, Tehran) were maintained under controlled conditions. All animals were acclimatized to the facility for 7 days prior to starting the experiments. Guidelines and ethical codes for laboratory animal care and handling was followed according to those set by the Iranian Ministry of Health and Medical Education.

All surgical procedures were performed under aseptic conditions. Following SCI surgery, three animals were housed per cage and maintained on a 12/12-h light/dark cycle, under controlled temperature (21 ± 2°C) and relative humidity (30–40%). Food and water were provided *ad libitum*.

### Spinal cord injury (SCI)

Traumatic spinal injury was induced with the weight-drop device developed at Baqiyatallah University (Gruner, 1992). Briefly, the rats (9–10 weeks old at time of injury; weight 225–275 g) were anesthetized with pentobarbital (40 mg/kg) administered intraperitoneally. The fur above the vertebral column was cleared using clippers, and cleaned with Betadine solution. A 20-mm midline incision was made in the thoracic region, and the vertebral column was exposed. A laminectomy was performed at the T10 vertebral level, exposing the dorsal cord surface with the dura remaining intact. The paravertebral muscle fascia was penetrated, and the muscles were peeled laterally using blunt dissection forceps. The spinal cord segment at the T8-T9 level was exposed by total laminectomy. The vertebral column was stabilized with angled clamps on the T8 and T12 vertebrae (Gruner, 1992; Basso et al., 1996). A 10 g weight (stainless steel rod, 3 mm diameter tip) was allowed to drop vertically from 12.5 mm (2.5 cm) onto the center of the exposed spinal cord, resulting in a moderate spinal cord injury (SCI). The impact rod was removed immediately following the injury, and the muscles and the incision were closed in layers using catgut. Following the surgery, the animals were placed on heating pads maintained at 37°C. The rats were monitored until they recovered from anesthesia, and were then returned to their home cages. The sham-operated rats received the same surgical procedures but sustained no impact injury (i.e., the spinal cord contusion was not performed).

### Plant collection and extraction

The plants were obtained from commercial sources. The dried leaf powder of *L. angustifolia* was macerated at room temperature in 70% ethanol (1 g/10 ml) and extracted for a week. On the seventh day, the ethanolic extract was filtered and the extract was evaporated under reduced pressure to remove the ethanol. The dry extract was suspended in normal saline. In this way, alcoholic extracts of *L. angustifolia* (Lav) at minimum (100 mg/kg), moderate (200 mg/kg), and maximum (400 mg/kg) doses were prepared.

### Drug treatments and experimental outline

The rats were divided into six groups as follows. Group I: intact (*n* = 6), group II: sham-operated/saline (*n* = 7), group III: control group subjected to SCI (*n* = 7), group IV: SCI treated with Lav 100 mg/kg (*n* = 7), group V: SCI treated with Lav 200 mg/kg (*n* = 10), and group VI: SCI treated with Lav 400 mg/kg (*n* = 8). Lav and saline, respectively, were injected intraperitoneally in the Lav and sham groups, starting 1 day after injury, then daily until the fourteenth day.

### Neurological examination of functional recovery

The Basso, Beattie, and Bresnahan (BBB) scale was used to assess neurological function in an open-field motor test. The BBB scale is a 21-point scale ranging from 1 (indicating no hindlimb movement) to 21 (indicating normal hindlimb function). It rates locomotion based on various aspects of hindlimb function, such as weight support, stepping ability, coordination, and toe clearance. All functional scores were obtained on post-injury days 1, 7, 14, 21, 28, 35, 42, 49, and 56 by two individuals blinded to treatment. Behavioral tests for the evaluation of pain were performed by means of the hot-water test for the hindlimbs before and after SCI; scores were obtained on post-injury days 1, 7, 14, 21, 28, 35, 42, 49, and 56.

### Electrophysiological evaluations

One day before the sacrifice of the animals, intramuscular EMG recording needles were inserted bilaterally into the hindlimb flexor muscles to record the 10-s electrical potential generated by the muscle cells. After recording, we compressed 10 to 1 s using EMG software in order to acquire the recruitment index of motor units.

### Histological preparation and morphometric studies

Eight weeks after the surgery and 1 day after electrophysiological evaluation, all rats were anesthetized (100 mg/kg sodium pentobarbital intraperitoneally), then were intracardially perfused with 0.9% saline followed by 10% buffered formalin. A 1 cm section of the spinal cord from the lesion's epicenter at T8 was dissected, post-fixed in 10% buffered formalin overnight and cryo-protected in 30% sucrose for 48 h. Serial 10 μm-thick sagittal sections were collected. All sections were processed for hematoxylin and eosin (H&E) staining and examined under light microscopy. The lesion area, including both the cavity and the surrounding damaged tissue, as well as gray and white matter volume and the number of lower motor neurons and positive GFAP astrocyte perikaryons, were then measured with image-analyzing software (Image-Pro Express, version 6.0.0.319, Media Cybernetics, Silver Spring, MD, USA). Three to five sections from each tissue specimen were counted, and mean values were obtained for each tissue. Only those cells that showed clearly discernible nucleus were counted. All cell-counting analyses were carried out by two observers blind to the specific experimental conditions of the analyzed tissues, on images acquired at 40 × and 400 × magnifications.

### Immunohistochemical analysis

Standard immunocytochemistry for the glial-scar GFAP was performed on all of the sections for each group. All cell-counting analyses were carried out on images acquired at 40 × and 400 × magnifications. The paraffin sections were dried in an air oven (58°C for 2~4 h. The tissues were dewaxed with xylene and hydrated with alcohol. After the sections were washed with PBS, antigen retrieval solution (0.01 mol/L sodium citrate, pH = 6.0) was added; then they were boiled at a constant temperature of 95°C 15 min. When the sections were naturally cooled down, PBS was used again to wash them. The tissues were incubated with H2O2 (3%) at room temperature for 10 min. They were washed with PBS, and 10% fetal calf serum was added for 30 min then the sections were washed with PBS thrice. Primary antibody (Abcam Plc, Cambridge, UK), namely anti-GFAP (1: 50), was added for the following incubation in humidified chamber at 4°C overnight. Avidin Biotin secondary antibody conjugated with HRP was added, incubation at 37°C was kept for 1.5 h. The samples were washed with PBS thrice. Finally DAB cromogen were used for 10 min and hematoxylin counterstaining (30 s) were successively performed. After dehydration and drying, the sections were mounted with Entelan. We used an ECLIPSE 5Oi microscope (Nickon ECLIPSE 5Oi Listed 4N75 inspection equipment 116284) to achieve better resolution for analyzing the images.

### Statistical analysis

All data, except for BBB and sensory function, were analyzed by one-way analysis of variance (ANOVA) followed by *post-hoc* Tukey and Dennett's multiple-comparisons tests. BBB and sensory function were analyzed by two-way ANOVA, followed by Bonferroni post-tests. The obtained data were presented as mean values ± standard error of mean (mean ± SEM); the treatment means were compared using the Tukey *post-hoc* test. A statistical probability of *P* < 0.05 was considered significant. All tests were performed using GraphPad Prism version 5.00 for Windows (GraphPad Software, San Diego, CA, USA).

## Results

### Effects of *L. angustifolia* extract on locomotor recovery after SCI

While SCI resulted in immediate paraplegia (loss of hindlimb movement), the sham-operated rats showed no significant changes in locomotion scores (BBB) in comparison with the intact animals. Intraperitoneal Lav administration (100, 200, or 400 mg/kg) 1 day after injury significantly improved locomotor function in the treated rats compared to the control group. Application of two-way ANOVA showed significant interactions between variables, such as Lav treatment (100, 200, and 400 mg/kg), and time [*F*_(40, 272)_ = 33.80, *P* < 0.0001].

**Figure 1:**
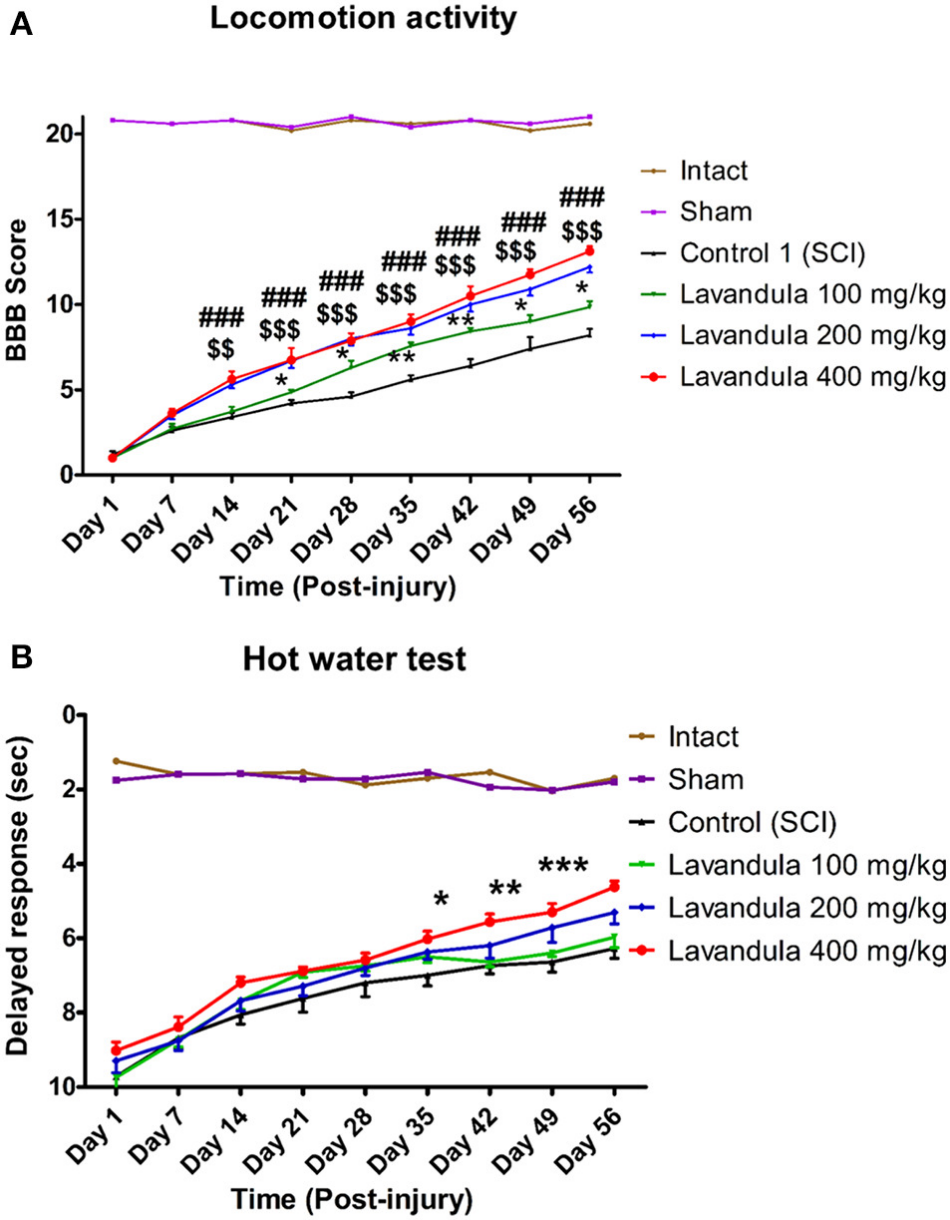
**Administration of ***L. angustifolia*** extract improves motor and sensory function impairment in the rat spinal cord contusion model**. Administration of *L. angustifolia* extract (i.p.) daily for 14 consecutive days post-injury significantly improved BBB scores **(A)** and sensory function (with decreased delayed response in the hot-water test) **(B)** Data are represented as mean ± SEM. **(A)** ###shows significant difference between Lav 400 and controls (SCI) (*P* < 0.001). $$ and $$$ show significant differences between Lav 200 and controls (SCI) (*P* < 0.01 and *P* < 0.001, respectively). ^*^ and ^**^show significant differences between Lav 100 and controls (SCI) (*P* < 0.05 and *P* < 0.01, respectively). (B)^*^, ^**^, and ^***^show significant differences between Lav 400 and SCI (*P* < 0.05, *P* < 0.01, and *P* < 0.001, respectively).

Application of the *post-hoc* Bonferroni multiple-comparisons test revealed significant improvement in motor function following Lav treatment on day 14 post-injury in the Lav 200 group (*p* < 0.01), and on days 21, 28, 35, 42, 49, and 56 in the Lav 400 group (*p* < 0.001). The Lav 100 group showed improved locomotor activity after day 28 (*p* < 0.05). There were significant differences between the doses of 100 and 400 mg/kg from day 14 (*p* < 0.05) until day 56 (*p* < 0.001). The results showed no significant differences between the Lav doses of 200 mg/kg and 400 mg/kg (Figure 1).

### Effects of *L. angustifolia* extract treatment on sensory recovery after SCI in the hot-water test

Statistical evaluations revealed that the mean latency time of response to painful stimulus (delayed response) was significantly decreased in the Lav groups vs. the control (SCI) group. Application of two-way ANOVA showed significant interactions between variables, such as Lav treatment (100, 200, or 400 mg/kg) and number of days post-injury [*F*_(40, 272)_ = 9.59, *P* < 0.0001]. Application of *post-hoc* Bonferroni's multiple-comparisons test revealed significant improvement in sensory function following Lav treatment on post-injury days 7, 14, 21, 28, 35, 42, 49, and 56. There was better sensory recovery with the highest Lav dose (400 mg/kg), but there were no significant differences between the other doses (Figure 1B). There were no significant differences between Lav 400 and Lav 200, or between Lav 100 and SCI, but there were significant differences between Lav 400 and the Lav 100 and SCI groups (*P* < 0.001).

### Electrophysiological evaluations

**Figure 2:**
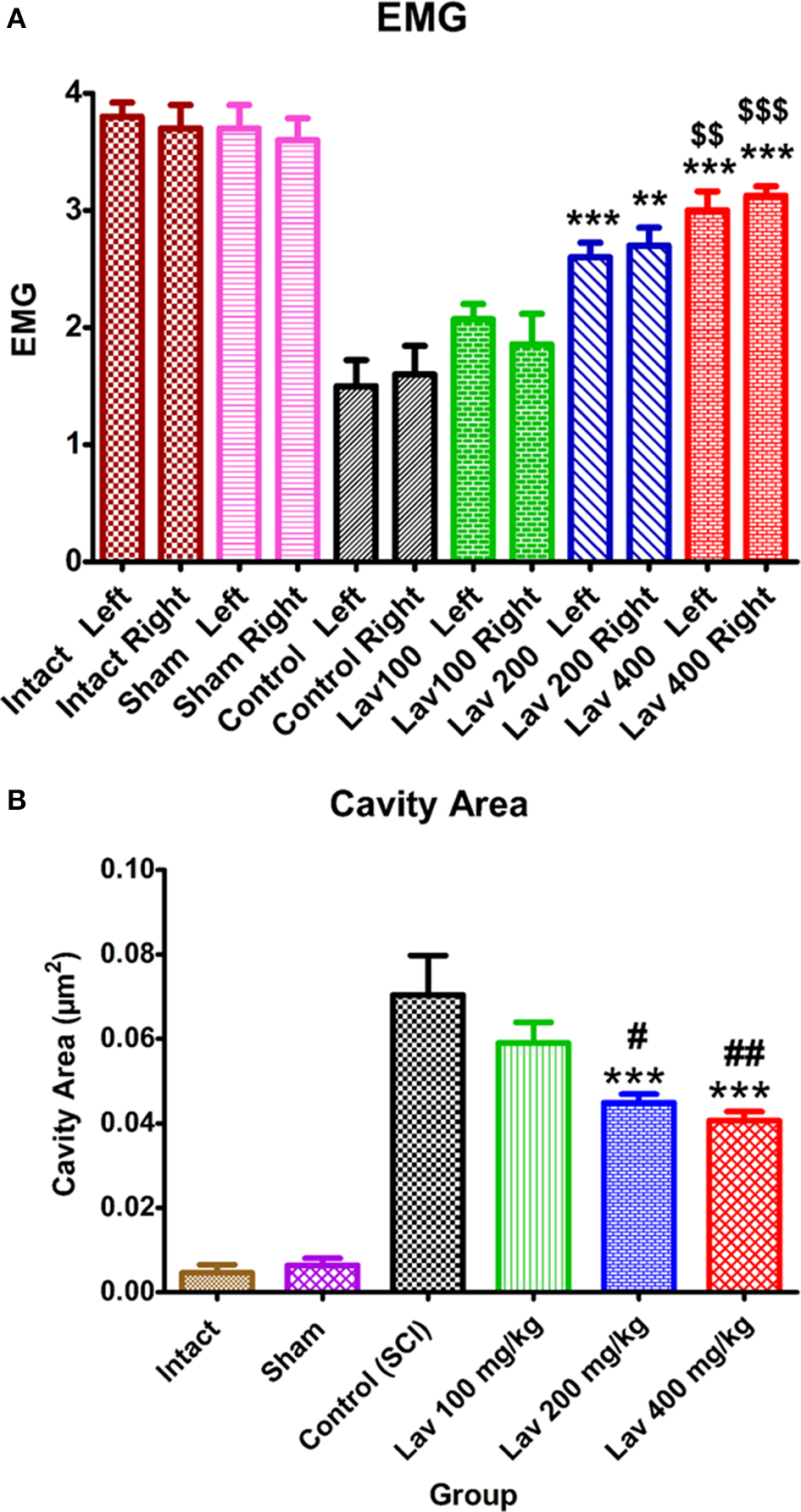
**Administration of ***L. angustifolia*** improved locomotor and EMG impairment in the rat spinal cord contusion model**. Intraperitoneal administration of *L. angustifolia* extract daily for 14 consecutive days post-injury significantly improved the EMG results **(A)** and histomorphological evaluations **(B)**. Data are represented as mean±SEM. **(A)** ^**^ and ^***^ show significant differences of the Lav 200 and 400 groups compared to the control group (SCI) (*P* < 0.01 and *P* < 0.001, respectively). $$ and $$$ show significant differences of the Lav 200 and 400 groups with the Lav 100 group (*P* < 0.01 and *P* < 0.001, respectively). **(B)** ^***^shows significant differences of the Lav 200 and 400 groups compared to the control group (SCI) (*P* < 0.001, respectively). # and ## show significant differences of the Lav 200 and 400 groups compared to the Lav 100 group (*P* < 0.01 and *P* < 0.001, respectively). (Per 35625 μm^2^).

Statistical analysis showed that the means of the recruitment index were increased significantly for the left [*F*_(5, 34)_ = 25.92, *P* < 0.0001] and right [*F*_(5, 34)_ = 18.17, *P* < 0.0001] hindlimbs in the Lav groups vs. the control (SCI) group. Application of Dennett's *post-hoc* multiple-comparisons test revealed significant improvements in electrophysiological activity of the right hindlimbs following treatment with Lav 200 mg/kg (*P* < 0.001) and 400 mg/kg (*P* < 0.0001). This test also revealed significant improvements in the electrophysiological activity of the left hindlimbs following Lav 200 and 400 mg/kg (*P* < 0.0001) treatments (Figure 2A). Although, there was some improvement with Lav 100 mg/kg in the right and left hindlimbs, there were no significant differences with the control group. There were significant differences between Lav 400 and Lav 100 in the left (*p* < 0.01) and right (*p* < 0.001) hindlimbs.

### Histomorphological evaluations

Statistical evaluations revealed that the mean cavity size was significantly reduced in the Lav 200 and Lav 400 treatment groups [*F*_(5, 34)_ = 37.32, *P* < 0.0001] compared to the control group (Figure 2B). No statistically significant difference was found in cavity volume between the Lav 200 and Lav 400 groups, but there were significant differences between Lav 100 and the Lav 200 group (*P* < 0.05) and the Lav 400 (*P* < 0.01) group.

**Figure 3:**
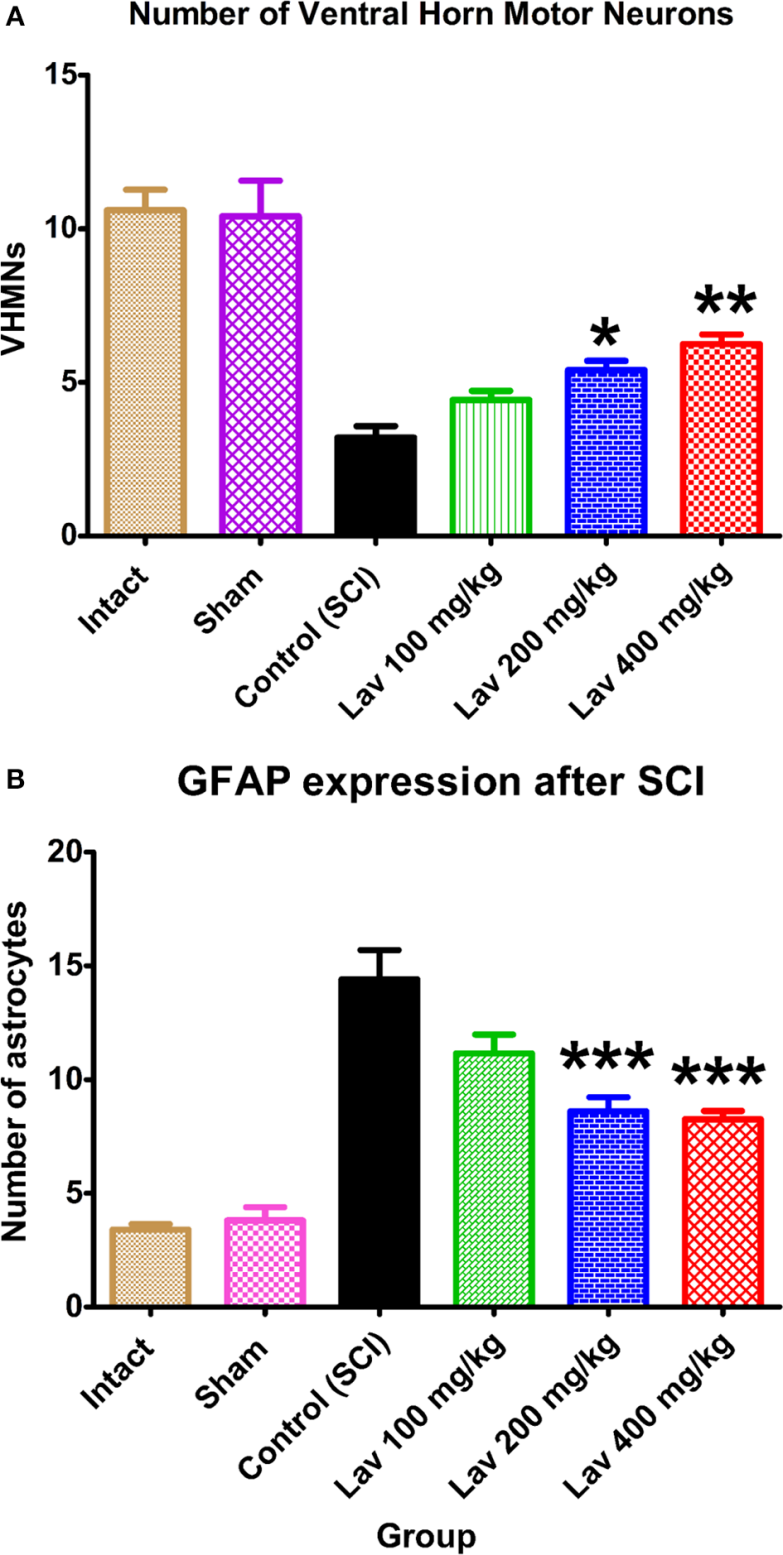
**Administration of ***L. angustifolia*** increased the number of VHMNs (A) and decreased GFAP expression in the rat spinal cord contusion model**. Intraperitoneal administration of *L. angustifolia* extract daily for 14 consecutive days post-injury significantly increased the number of VHMNs **(A)** and decreased the levels of GFAP expression in the rat spinal cord contusion model **(B)**. Data are represented as mean ± SEM. **(A)** ^*^ and ^**^ show significant differences of the Lav 200 and Lav 400 groups with the control (SCI) group (*P* < 0.05 and *P* < 0.01, respectively). (Per 5700 μm^2^). **(B)** ^***^ shows significant differences of the Lav 200 and Lav 400 groups with the control (SCI) group (*P* < 0.001). (Per 35625 μm^2^).

Application of one-way ANOVA showed significant differences between the sham, Lav-treated, and control groups in the number of ventral horn motor neurons (VHMNs) [*F*_(5, 34)_ = 30.73, *P* < 0.0001]. Application of Dunnett's *post-hoc* multiple-comparisons test revealed a significant increase in the number of VHMNs in the Lav 200 (*p* < 0.05) and Lav 400 (*p* < 0.01) treatment groups compared to the control group (Figures 3A, 5). No significant differences were observed between Lav 100 and Lav 200.

### GFAP expression after SCI

**Figure 4:**
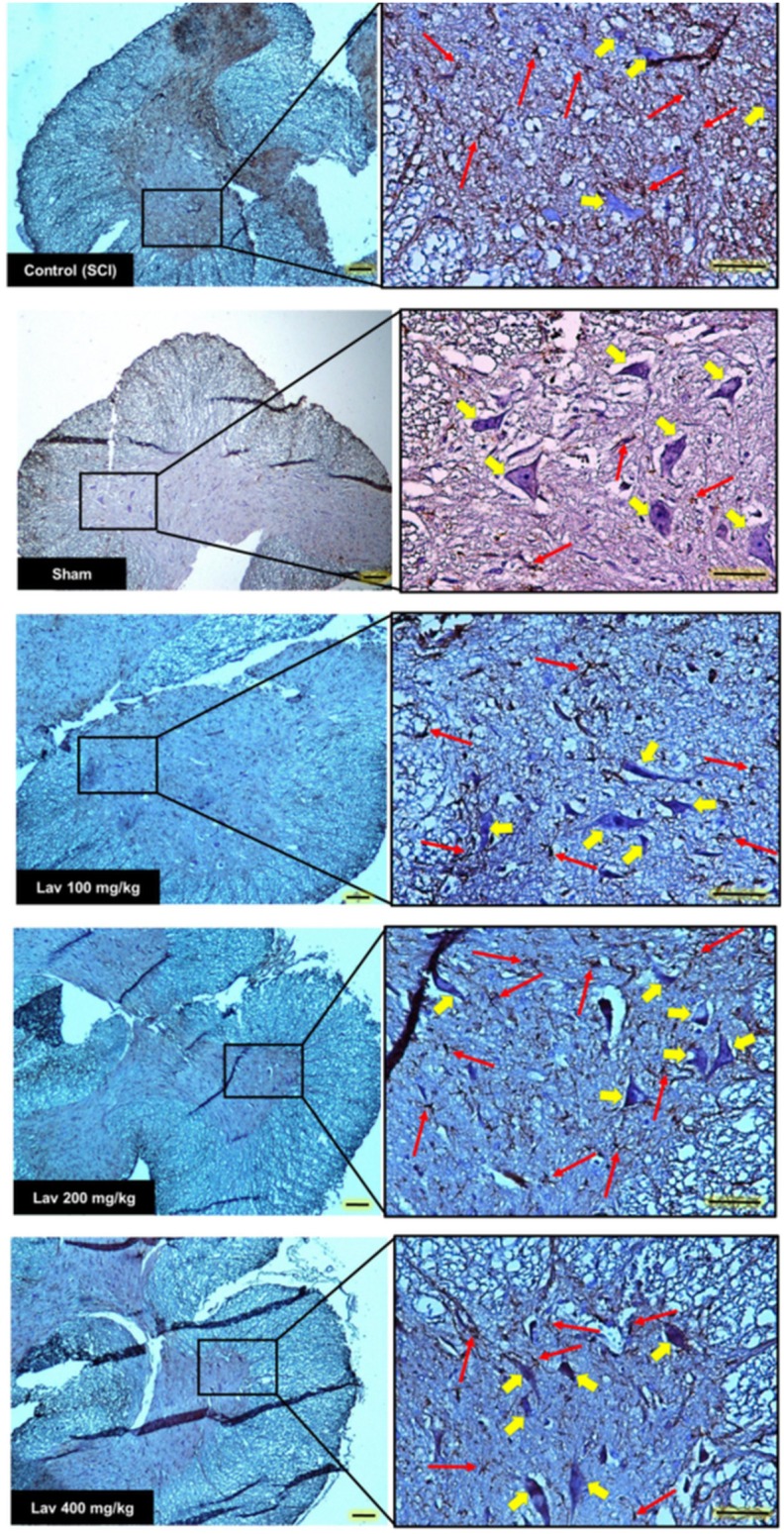
**Transverse section of spinal cord showing the ventral horn gray matter at the T12-L1 level for all groups on day 56 GFAP-stained images**. Red arrows indicate the GFAP astrocytes. Yellow arrows indicate VHMNs. Decreased GFAP astrocytes and increased VHMNs are evident. Bar in 40X = 100 micrometer and Bar in 200X = 50 micrometer. (ECLIPSE 5Oi microscope).

Strong immunostaining for GFAP was demonstrated in the control group (Figures 3B, 4); however, this activation was significantly attenuated in the Lav groups [*F*_(5, 34)_ = 28.18, *P* < 0.0001]. Application of Bonferroni's *post-hoc* multiple-comparisons test revealed a significant decrease in GFAP in the Lav 200 and Lav 400 groups (*p* < 0.001; Figure 3B). These results revealed that Lav can abate the activation of astrocytes after SCI. No statistically significant difference was found in GFAP between the Lav 100, Lav 200, and Lav 400 groups.

**Figure 5:**
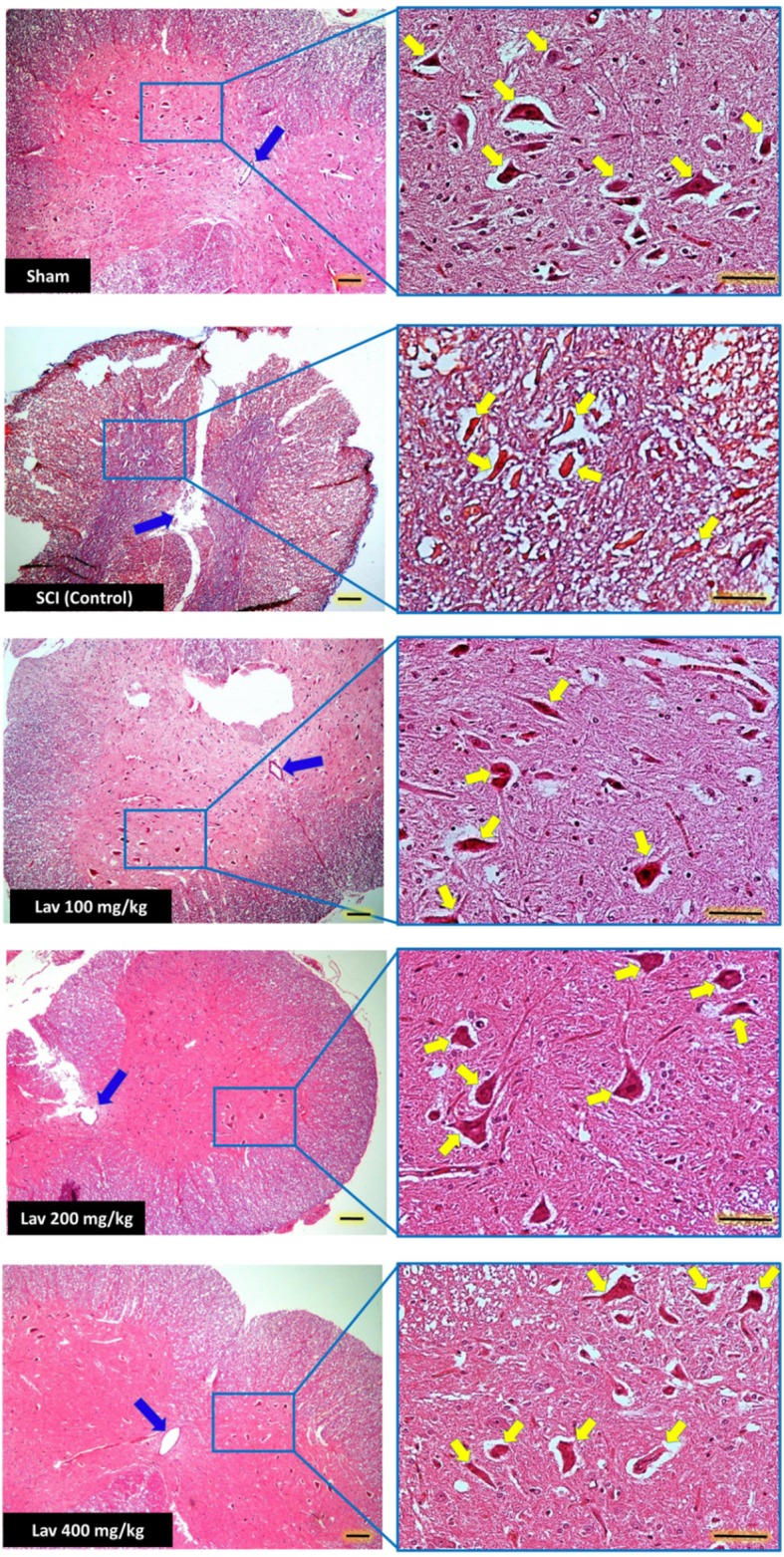
**Administration of ***L. angustifolia*** improved Histomorphological evaluation results in the rat spinal cord contusion model**. Transverse section of spinal cord showing the ventral horn gray matter from spinal cord at the level of T12-L1 of all groups which evaluated in this study at day 56. H&E staining showed shrinkage and decrease of Ventral Horn Motor Neurons of control (SCI) in compare with Lav 100, 200 and 400 mg/Kg/Day. Yellow arrows illustrating the Ventral Horn Motor Neurons and blue arrows show central canal. *L. angustifolia* extract increased the number of VHMNs and improved the shape of central canal. Bar in 40X = 100 micrometer and Bar in 200X = 50 micrometer. (ECLIPSE 5Oi microscope).

## Discussion

*L. angustifolia* has neuroprotective and neurotrophic effects, including enhancement of functional recovery (Vakili et al., 2013), suggesting that it has therapeutic effects on neurodegenerative disease (Peana et al., 2003, 2006; Vakili et al., 2013) and antinociceptive effects on SCI (Peana et al., 2004). We evaluated the therapeutic potential of *L. angustifolia* for SCI in a conventional animal model. Intraperitoneal *L. angustifolia* improved motor dysfunction following that administration of *L. angustifolia* extract itself improves the behavioral and cellular outcomes in the rat contusion SCI model.

SCI is a complex neuropathological process involving a variety of neurochemical, cellular, and molecular events. Calcium overload (Dumont et al., 2001), extracellular accumulation of glutamate (Beattie et al., 2002), and induction of oxidative stress (Azbill et al., 1997; Aksenova et al., 2002) are the results of the primary injury to the spinal cord after the initial trauma. Increased oxidative stress after spinal cord trauma can lead to secondary processes, such as impaired activity of membrane enzymes (Aksenova et al., 2002; Martin and Liu, 2002) and over-expression of inflammatory mediators, which potentiates secondary injury to the spinal cord via a variety of processes, such as activation of microglia and stimulation of astrocyte proliferation, which in turn can even further increase the generation of neurotoxic ROS (Dumont et al., 2001; Popovich and Jones, 2003).

It has been demonstrated that administration of *L. angustifolia* extract could alleviate the extracellular accumulation of glutamate (Büyükokuroğlu et al., 2003) and could decrease oxidative stress (Peana et al., 2006; Vakili et al., 2013), and it has reported that linalool (a constituent of *L. angustifolia*) has antinociceptive effects (Peana et al., 2003).

Ideally, therapeutic strategies for SCI would incorporate both neuroprotective and neurotrophic properties. Acute neuroprotection could preserve neurologic function by maintaining axonal function and preventing cell death. Ionic flux, alterations in local blood flow, and inflammation contribute to the initial and evolving secondary neurologic injuries, and therapeutic strategies have attempted to reduce excitotoxicity with glutamate receptor antagonists (Rosenberg et al., 1999; Wada et al., 1999) and to inhibit nitric oxide synthase (Satake et al., 2000), block apoptosis (Schumacher et al., 2000), and modulate inflammatory responses (Schwartz et al., 1999).

Protection against the progression of secondary injury to the spinal cord neurons appears to be one of the most effective therapeutic strategies for limiting tissue injury and improving the outcome of spinal cord trauma (Dumont et al., 2002). Lav improved locomotion and increased VHMNs; therefore, it appears that it could play an important role in treatment strategies. As neuroprotection could preserve neurologic function by preventing cell death, one of the most important roles of Lav could be its neuroprotective effects. We believe that calcium-calmodulin may play an important role in the neuroprotective effects of Lav (Kayvan Yaghoobi et al., 2015).

## Conclusion

A non-immunosuppressive *L. angustifolia* extract improved motor dysfunction in a SCI contusion model, and promoted morphological improvement. While further studies are needed to clarify the mechanism of action in SCI models, the present results suggest that *L. angustifolia* extract may have therapeutic potential for treating patients with spinal cord injuries.

## Author contributions

KY developed the original idea and the protocol, abstracted and analyzed the data, wrote the manuscript, and acted as guarantor. GK, SH, SD, SS, and KM contributed to the development of the protocol, abstraction of data, and preparation of the manuscript. GK supervised the protocol.

### Conflict of interest statement

The authors declare that the research was conducted in the absence of any commercial or financial relationships that could be construed as a potential conflict of interest.
